# Towards a neurophysiological model of kundalini: a theoretical framework informed by preliminary clinical observations

**DOI:** 10.3389/fnbeh.2026.1828520

**Published:** 2026-06-10

**Authors:** Swapan Samanta, Nirmal Sultania, Mukul Roychoudhury, Surendra Sharma, Paritosh Mitra

**Affiliations:** INDORIV Clinical Research Centre, Kolkata, India

**Keywords:** kundalini, autonomic integration, consciousness, contemplative neuroscience, heart rate variability, neural dispersion index, liberation phenomenology, dharana

## Abstract

The purpose of the present paper is not to validate the metaphysical claims of yogic traditions, but to examine whether their phenomenological descriptions correspond to measurable physiological processes. The proposal advanced here is explicitly theoretical: a hypothesis-generating model informed by preliminary retrospective clinical observations, not a claim of established proof. All interpretations of iconographic material are advanced as phenomenological correspondences — possible mappings onto identifiable neurophysiological states — rather than assertions of historical or archaeological fact.

**Background:**

Kundalini has been described in yogic literature as a transformative psychophysiological process, with systematic iconographic representations that date back across millennia. Its physiological basis remains undefined within contemporary neuroscience. This article proposes that the phenomenological descriptions embedded in classical Indian iconography may correspond in structure and sequence to identifiable neurophysiological states of autonomic integration.

**Objective:**

This article proposes a neurophysiological theoretical framework for interpreting kundalini phenomena, drawing on preliminary, retrospective clinical observations, published neuroscience, and cross-tradition phenomenological analysis.

**Methods:**

A retrospective analysis of a 14-year single-practitioner observational dataset comprising 404 consecutive patients treated for autonomic dysregulation, sleep disturbance, and attentional dysfunction using non-invasive, non-pharmacological interventions. No randomisation or control arm was employed. The dataset is presented as exploratory and hypothesis-generating clinical evidence rather than experimental proof.

**Results:**

Observed patterns—including improvements in heart rate variability (HRV), cortisol diurnal rhythm restoration, and attentional stability—are associated with a progressive nervous system integration trajectory consistent with the classical bottom-up kundalini model. The neural dispersion index (NDI), proposed in this study as an exploratory heuristic synthesis metric requiring independent validation, is used to track these changes across a seven-domain profile. The natural clinical progression follows a four-stage sequence: fragmentation (NDI > 60) → dormant baseline (NDI 40–60) → progressive integration (NDI 25–40) → threshold coherence (NDI < 25).

**Conclusion:**

Preliminary observations support a testable neurophysiological model of kundalini as a process of progressive autonomic and cortical integration. The model generates four specific falsifiable predictions. Formal prospective investigation is required before any clinical conclusions can be drawn.

## 1 Introduction

A recurrent observation across clinical practice is that a substantial category of human suffering resists the diagnostic categories available to address it. Patients presenting with autonomic dysregulation, attentional instability, and sleep architecture disruption frequently show normal laboratory values and unremarkable imaging but report persistent functional impairment. This clinical gap—between measurable pathology and experienced distress—motivates this investigation.

This study addresses the clinical gap by drawing on a source that biomedical researchers rarely consult: systematic phenomenological observations embedded in ancient contemplative iconography. Specifically, it examines the Shiva-serpent iconographic complex of classical Indian tradition, not as a religious artefact requiring theological interpretation, but as a candidate phenomenological map that may correspond to what contemporary neuroscience recognises as the human autonomic, neuroendocrine, and cortical integration systems.

The proposal is not that ancient practitioners possessed neuroimaging technology, but rather that systematic first-person observation of the human organism, conducted with rigour over centuries across multiple traditions, may have converged on the same functional architecture that modern neuroscience has mapped from the outside. The degree to which this correspondence holds is precisely the hypothesis this framework is designed to generate and test.

Several bodies of convergent evidence provide the theoretical scaffolding for this approach. Porges’s polyvagal theory has established a hierarchical organisation of the autonomic nervous system along an evolutionary axis that structurally maps the bottom-up integration sequence described in yogic literature (Porges, 2011). Research on heart rate variability as an integrative biomarker of nervous system coherence has provided measurable proxies of states that contemplative traditions described phenomenologically (Thayer and Lane, 2009). Neuroimaging research on long-term meditators has documented structural and functional changes—increased prefrontal grey matter density, reduced amygdala reactivity, and altered default mode network activity—that are broadly consistent with the integration model proposed in this article (Hölzel et al., 2011; Brewer et al., 2011).

### 1.1 Prior neurophenomenological approaches to kundalini

Several earlier attempts to bridge kundalini phenomenology and neuroscience warrant acknowledgement. Sanches and Daniels (2008) developed the Kundalini Awakening Scale (KAS), a 76-item psychometric instrument capturing five experiential subscales. The KAS represents the most systematic prior effort to quantify kundalini-related subjective experience, although it operates without an underlying neurophysiological model that links experiential reports to measurable biomarkers. Woollacott et al. (2020) documented the phenomenology, physiology, and transformative impact of kundalini awakenings in a large sample. Kotiyal et al. (2022) characterised sensory, motor, and affective features of kundalini-related experiences during tantric yoga meditation, documenting ascending somatic sensations consistent with the bottom-up integration trajectory proposed in this model. Jha et al. (2023) offered a neuroscientific perspective on kundalini as a form of psychobiological arousal, and Yadav et al. (2024) provided a neurodynamic analysis of spinal energy during various breathing techniques.

Earlier proposals mapping chakras onto anatomical nerve plexuses have also appeared in the literature (e.g., Mukherjee and Saha, 2023). This article acknowledges these prior mappings and distinguishes its contribution as fourfold: (Berkovich-Ohana et al., 2012) embedding the anatomical correspondence within a three-state integration model derived from iconographic analysis; (Bowlby, 1988) *while the anatomical mapping itself is not new, the present article assigns station-specific candidate biomarkers to each proposed plexus station, thereby creating a multi-domain, measurable framework for tracking integration sequentially through the system*; (Brewer et al., 2011) proposing a composite heuristic metric (NDI) for tracking integration across the full sequence; and (Brown and Gerbarg, 2005) providing a mathematical formalism for the endpoint of the integration process, which is currently accepted for publication as a companion article (Samanta, 2026, *Frontiers in Psychology*).

Three specific gaps in the existing literature motivate this study: the absence of a multi-domain composite biomarker for tracking integration across the full kundalini trajectory; the absence of a mathematical formalism enabling a cross-tradition comparison of liberation phenomenology; and the absence of a theoretical framework linking the iconographic sequence to a testable neurophysiological model with specific falsifiable predictions.

This article makes four primary contributions: first, a theoretical neurophysiological reinterpretation of the three-state Shiva-serpent iconographic model; second, while prior literature has proposed anatomical correspondences between chakra positions and nerve plexuses, this article assigns station-specific candidate biomarkers to each proposed plexus station, creating a multi-domain measurable framework for tracking integration sequentially through the system; third, it reports preliminary retrospective clinical observations from a 14-year single-practitioner dataset; and fourth, a mathematical operator—the R-Operator—describing the structural principle common across five major contemplative traditions’ accounts of liberation, developed in full in the companion article (Samanta, 2026).

#### 1.1.1 Evidence classification

Throughout this article, three evidence tiers are distinguished. Claims designated [MEASURED] are supported by peer-reviewed literature or the observational cohort data. Claims designated [CLINICALLY OBSERVED] are systematically documented across the patient cohort (*n* = 404; documented in contemporaneous clinical records, 2010–2024; see Appendix A for observational protocol) but have not been RCT-validated. Claims designated [THEORETICAL MODEL] are formally proposed frameworks requiring prospective investigation.

## 2 Study design and ethics

This article presents a theoretical neurophysiological framework informed by a retrospective observational dataset. The observational cohort comprises 404 consecutive patients treated in a single-practitioner clinical setting between 2010 and 2024. No randomisation, blinding, or control arm was employed. The observational cohort is presented as exploratory, hypothesis-generating evidence supporting a theoretical neurophysiological model.

Ethics approval for the broader research programme was granted by the institutional ethics committee (HP/EC/APVL/20-24/058.3). Written informed consent was obtained from all adult participants; assent was obtained from minor participants with parental or guardian consent. The study was conducted in accordance with the Declaration of Helsinki. All clinical findings—biomarker frameworks (NDI), intervention protocols (Attachment-Based Sleep Therapy (ABST), The Recursive Self-Inquiry Protocol (RSIP), 8-week integration programme), and theoretical models (neural restoration hypothesis and R-Operator)—reported in this study are original contributions of this research programme unless otherwise attributed.

The mathematical formalism for liberation phenomenology (R-Operator) referenced in this article is developed in full in the companion article: Samanta S. “Liberation Mathematics I: The Behavioral and Consciousness Definition of Perpetual Self-Transcendence,” accepted for publication in *Frontiers in Psychology* (section: Theoretical and Philosophical Psychology; Research Topic: Advances in Contemplative Science—Volume II; Manuscript ID: 1741096).

## 3 The Shiva-serpent iconographic system: a phenomenological reading

### 3.1 Beyond theology: the Iconogram as candidate phenomenological map

For readers unfamiliar with the visual conventions of classical Indian iconography, a brief orientation is helpful. The Shiva-serpent iconographic complex is among the most ancient and widely distributed image systems in South Asian visual culture. Across thousands of depictions spanning more than two millennia—in stone sculpture, bronze casting, and painted manuscript—the deity Shiva is consistently shown in relation to a serpent (identified in the textual tradition as Vāsuki). The serpent’s position relative to the deity’s body varies systematically across different iconographic forms, and it is this positional variation that this analysis examines. Supplementary Figure S1 provides representative visual references for the three positional states discussed.

The Natārāja and Dakshiṇāmūrti forms of Shiva consistently depict a serpent in one of three positional states relative to the deity’s body: coiled at the base of the spine, raised with head erect, or worn loosely at the neck as an ornament with a visible gap between serpent and skin. Classical commentators have interpreted this serpent as Vāsuki (cosmic serpent), as kuṇḍalinī śakti (dormant energy), or as a symbol of time and ego-dissolution.

**Figure 1:**
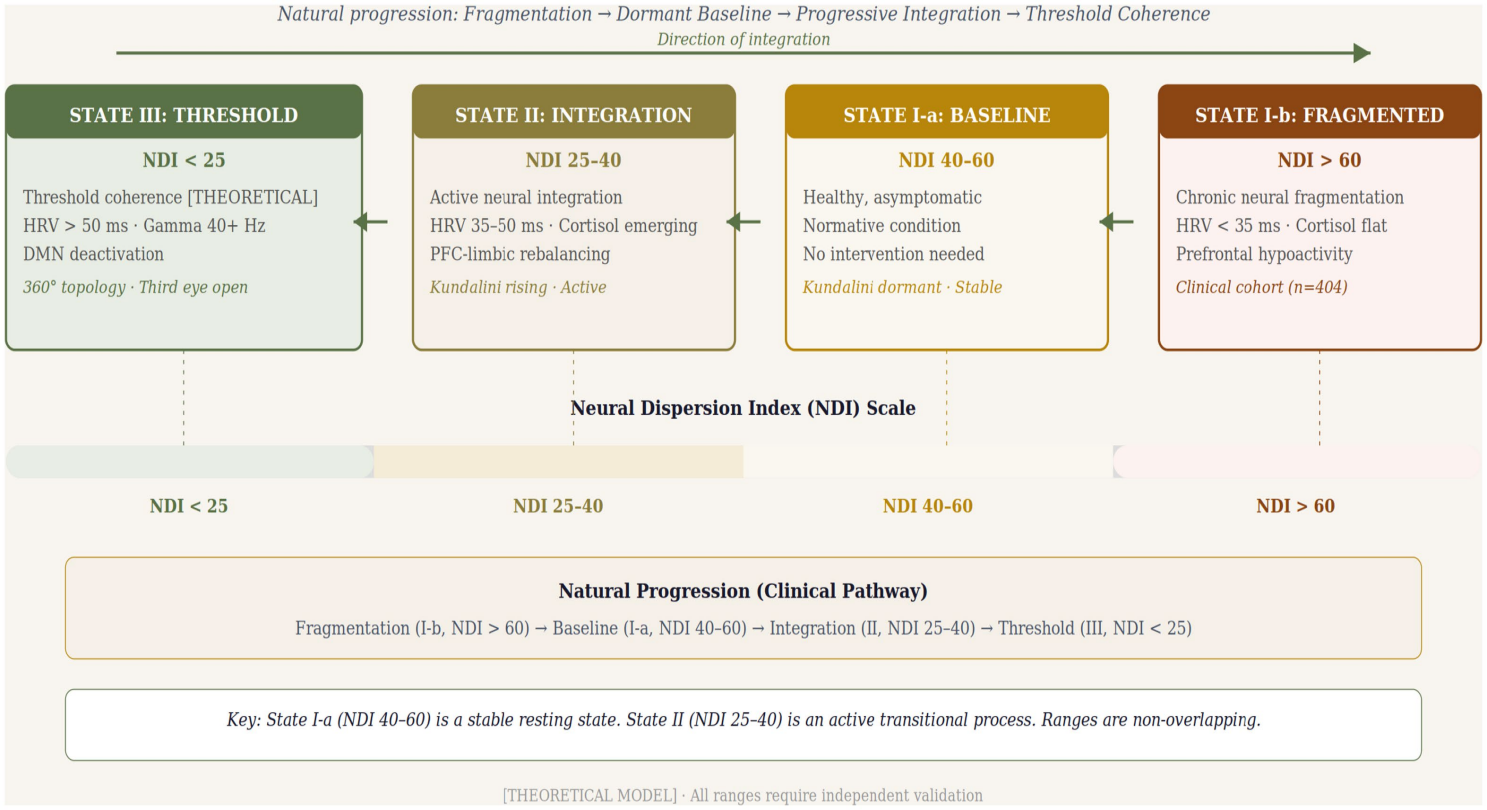
Four-stage neurophysiological integration model revised three-state model of nervous system organisation derived from Shiva-serpent iconographic analysis. Four-stage model with non-overlapping NDI ranges: State I-b (fragmented, NDI > 60), State I-a (dormant-baseline, NDI 40–60), State II (progressive integration, NDI 25–40), State III (threshold coherence, NDI < 25). Natural clinical progression: Fragmentation → Baseline → Integration → Threshold. State I-a is a stable resting state; State II is an active transitional process. NDI = Neural Dispersion Index (proposed heuristic metric; see Section 4). All state designations are theoretical correspondences. The liberated state (State III) encodes a shift in mode of identification, not ontological separation. [THEORETICAL MODEL].

**Figure 2:**
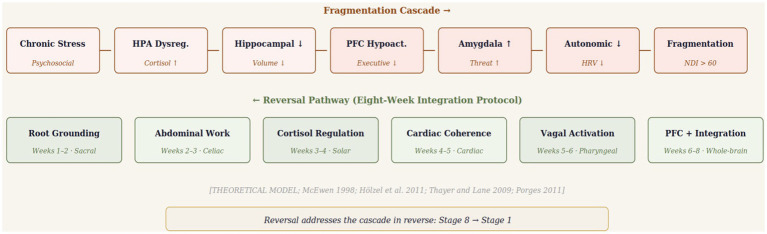
Proposed neural fragmentation cascade with reversal pathway proposed eight-stage neural fragmentation cascade with bottom-up reversal pathway. The reversal pathway addresses the cascade in reverse sequence through plexus-targeted intervention. [THEORETICAL MODEL; McEwen, 1998; Hölzel et al., 2011; Thayer and Lane, 2009; Porges, 2011].

A neurophysiological reading proposes something more specific: that these three positions may encode three observable states of nervous system organisation and that the iconographic sequence as a whole may constitute a procedural phenomenological map—what we might tentatively call, in contemporary terms, a clinical algorithm—for the progression between these states. This reading is offered as a hypothesis for investigation, not as a claim about the original intent of the iconographic tradition (Figures 1, 2 and Tables 1, 2).

**Table 1:** Proposed plexus-chakra mapping with candidate biomarkers.

Chakra station	Plexus	Neuroendocrine	Function	Biomarker
1—Mūlādhāra (Root)	Sacral (S2–S4)	Serotonin · GABA	Pelvic floor · Somatic safety	Gut serotonin · Pelvic EMG
2—Svādhiṣṭhāna (Sacral)	Hypogastric (L1–L2)	DHEA · Gonadal	Reproductive regulation	DHEA/cortisol ratio
3—Maṇipūra (Solar)	Celiac (T5–T12)	Cortisol · Adrenaline	HPA axis · Gut-brain	Cortisol rhythm · Insulin
4—Anāhata (Heart)	Cardiac (T1–T5, X)	Oxytocin · BDNF	Vagal tone · Attachment	HRV (RMSSD) · Cardiac coherence ratio
5—Viśuddha (Throat)	Pharyngeal (IX, X, XI)	T3/T4 · Calcitonin	Thyroid · Upper vagal	Thyroid profile · Nostril dominance laterality
6—Ājñā (Third Eye)	PFC–Pineal axis	Melatonin · Dopamine	Executive function · Sleep	Alpha coherence · PFC
7—Sahasrāra (Crown)	Whole-brain (DMN)	Gamma (40+ Hz)	Global synchrony · Non-dual	Gamma · DMN deactivation

Proposed mapping of seven chakra stations onto candidate anatomical nerve plexuses. Revised biomarkers include cardiac coherence ratio at Anāhata (McCraty et al., 2009) and nostril dominance laterality at Viśuddha (Śiva Svarodaya; Telles et al., 1994). All correspondences are proposed theoretical mappings requiring empirical validation. [THEORETICAL MODEL].

**Table 2:** Proposed neural dispersion index (NDI): seven-domain framework.

Domain	Weight	Proxy	Fragmented	Transitional	Integrated
Autonomic	15%	HRV (RMSSD)	<35 ms	35–50 ms	>50 ms
Neuroendocrine	15%	Cortisol Diurnal	Flat (AM=PM)	Mild slope	Clear AM peak
Attentional	15%	Sustained Focus	<3 min	3–8 min	>12 min
Somatic	10%	Body Coherence	High pain/dissoc.	Moderate	Stable, integrated
Affective	15%	Emotional Reg.	Reactive, labile	Moderate stab.	Responsive, stable
Cognitive	15%	PFC Engagement	Hypoactive	Moderate	Sustained exec.
Interpersonal	15%	Social Coherence	Disconnection	Variable	Secure, attuned

Proposed model NDI: seven-domain heuristic framework. NDI > 60 = fragmentation (State I-b); NDI 40–60 = normative baseline (State I-a); NDI 25–40 = active integration (State II); NDI < 25 = threshold coherence (State III). Ranges are non-overlapping. An exploratory instrument requiring independent validation. [THEORETICAL MODEL].

The key observational feature in the liberated state (Dakshiṇāmūrti) is a constellation of precisely coordinated iconographic elements. The deity’s body is depicted in corpse-like stillness—what the tradition itself calls the *śava*-state (literally: “corpse”; cf. the classical aphorism “Shiva without Shakti is *shava*”). The two physical eyes are closed or half-closed, indicating cessation of ordinary sensory engagement. The third eye (*ājñā*) is depicted as open—the only element that is open when everything else has closed. The face carries the trace of a smile, and the serpent (Vāsuki) is worn loosely around the neck in a 360-degree loop with a visible gap between the serpent and the skin: present, alert, yet neither constricting nor fused.

The phenomenological proposition encoded in this image-complex is precise: When the body’s sensory apparatus ceases (eyes closed, corpse-state), a different mode of witnessing persists that was never dependent on the sensory apparatus (third eye open). Consciousness undergoes a shift in its mode of identification with the body–mind system—from positional fusion to non-positional witnessing—while remaining fully present, aware, and functional within and through the body. This formulation avoids the ontological dualism that would be implied by a claim of “separation”; it describes, rather, a reconfiguration of the relationship between consciousness and its somatic substrate, consistent with non-dual frameworks (cf. the Advaita distinction between *vivartavāda*—apparent transformation—and ontological separation).

An important contextual clarification is warranted in this article. The classical tradition understood the neurological, psychological, and somatic benefits of yogic practice as *secondary consequences* of a transformation whose primary aim is liberation (*mokṣa*)—the irreversible shift in identification described above. The neurophysiological correlates examined in this article are, from the tradition’s own perspective, accompaniments of the liberative process rather than its purpose. This framework’s contribution is to propose that these accompaniments may be measurable, trackable, and clinically relevant—without displacing the tradition’s own understanding that the physiological benefits follow from, and are subordinate to, the shift in consciousness itself.

### 3.2 The three states: phenomenological and physiological correspondence

State I—Kundali Shayita—requires a critical distinction. The dormant (*śayita*) state of kundalini is the *normative condition of the overwhelming majority of human beings*—including those leading healthy, productive, and emotionally stable lives. Dormancy is not pathology. A person with dormant kundalini may have excellent health, strong relationships, professional success, and psychological stability. The tradition’s own framework is clear on this point: the *śayita* state is the default condition, not a disease state.

State I-a: Dormant-Baseline. Kundalini is dormant. The nervous system operates within normal functional parameters. The individual may be healthy, well-adjusted, and asymptomatic. NDI in this sub-state may range from 40 to 60 (the normative range for most adults). No clinical intervention is indicated. Individuals in State I-a may *choose* to pursue contemplative practices for the purpose of liberation (*mokṣa*)—but that is a philosophical decision, not a clinical one.

State I-b: Dormant-Fragmented. Kundalini is dormant, *and* the nervous system has undergone the fragmentation cascade described in Section 4.2—chronic stress exposure, hypothalamic–pituitary–adrenal (HPA) axis dysregulation, prefrontal hypoactivity, and sleep disruption. NDI > 60. Clinical intervention is indicated. This is the sub-state that this article’s clinical cohort (*n* = 404) primarily represents. The metaphor of a serpent coiled tightly, head pressed downward, may represent this fragmented condition (McEwen, 1998). Epidemiological context is relevant: Approximately 40 million American adults carry formal anxiety diagnoses, sustained attentional duration has declined substantially, and sleep architecture disruption affects roughly one-third of the industrialised adult population (van der Kolk, 2014).

The critical clarification is that *awakening (State II) is not the treatment for State I-a*. A person in the dormant-baseline condition does not need clinical intervention. The integration protocol described in this article is designed for individuals in State I-b whose nervous system has been disrupted by pathological fragmentation.

State II—Kundali Jagrita—corresponds to functional neural integration: the progressive bottom-up reactivation of candidate plexus stations characterised by increasing HRV, restoration of cortisol diurnal rhythm, and improved prefrontal regulatory capacity. State II corresponds to NDI 25–40—a range distinct from and below the normative baseline (State I-a, NDI 40–60), reflecting the active reorganisation that occurs when the nervous system moves beyond its resting parameters through sustained practice. The modern wellness industry—yoga studios, meditation apps, and breathwork programmes—appears to address this transitional state, generally without articulating a theoretical framework that explains when and why its interventions are effective (Streeter et al., 2010; Brown and Gerbarg, 2005).

State III—Kundali Mukta—is the most epistemically demanding component of the framework. It is presented in this article as a theoretical hypothesis informed by (a) consistent cross-tradition phenomenological descriptions across traditions operating within substantially different frameworks—including traditions between which documented historical exchange was limited or indirect; (b) emerging neuroimaging research on non-dual awareness and DMN deactivation; (c) high-amplitude gamma synchrony documented in long-term meditators; and (d) first-person observational evidence offered explicitly as phenomenological data rather than clinical proof.

## 4 Proposed anatomical mapping: seven plexuses as candidate integration stations

The seven-chakra system, when examined independently of its energetic and theological interpretive overlay, resolves into a candidate map of the human peripheral and central nervous system’s major integration nodes. Each chakra location corresponds to a documented anatomical plexus with identified neurotransmitter profiles, endocrine connections, and measurable functional biomarkers. The correspondence proposed in this article is not asserted as proof; it is offered as a structural hypothesis worthy of systematic empirical investigation. Prior proposals mapping chakras onto anatomical plexuses have appeared in the literature (Mukherjee and Saha, 2023); the present contribution embeds this mapping within a three-state integration model with station-specific biomarkers and a composite tracking metric.

An epistemological clarification is required in this article regarding the Pañcakośa (five-sheath) model. The classical framework, first systematically expounded in the *Taittirīya Upaniṣad* (Brahmānandavallī, II.1–5; Taittirīya Upaniṣad, 1957) and elaborated in Śaṅkarācārya’s *Vivekacūḍāmaṇi* (verses 149–209; Śaṅkarācārya, 1921; see also Feuerstein, 1998), describes kundalini and chakra phenomena as belonging to the Prāṇamayakośa (vital-energy sheath), which is ontologically distinct from the Annamayakośa (physical-body sheath) in the traditional system. This article’s epistemological position is to examine whether the phenomenological descriptions embedded in the classical system correspond to measurable physiological processes, without adjudicating the ontological claims of the tradition. The proposed chakra-to-plexus mapping is a translation across kośa boundaries: It takes descriptions assigned to the Prāṇamayakośa level and asks whether the functional states they describe have identifiable correlates at the physical level. We do not claim that Prāṇamayakośa *is* the autonomic nervous system. We propose that the functional states described at the Prāṇamayakośa level may *correspond to* measurable states at the physical level and that testing this correspondence is a legitimate empirical research programme.

### 4.1 The celiac plexus: an underutilised clinical target

Of particular clinical interest is the celiac plexus—the candidate Manipura station. The enteric nervous system, which the celiac plexus dominates, contains approximately 500 million neurons and is capable of independent regulation and bidirectional communication with the central nervous system (Mayer, 2011). Gut serotonin accounts for approximately 95% of the body’s total serotonin production. Cortisol dysregulation, the most prevalent biomarker of chronic fragmentation in the observational cohort, is directly modulated through celiac plexus-mediated HPA axis activity (Pert, 1997).

The yogic practice of Uddiyana Bandha—abdominal retraction on exhaled breath—applies direct mechanical pressure to the celiac plexus region and is associated with what appears to be measurable vagal activation (Jerath et al., 2006). This interpretation of what classical texts described as “energy locking” as direct autonomic plexus stimulation is one of the framework’s most practically applicable clinical proposals.

### 4.2 The tongue as trigeminal–vagal Interface

A less-discussed anatomical feature of the classical system is the disproportionate attention given to the tongue in practices such as Khechari Mudra (tongue-to-palate contact). Neuroanatomical evidence supports a functional basis: The tongue occupies the largest cortical representation of any body part in the sensory homunculus and is the convergence point of cranial nerves V (trigeminal) and XII (hypoglossal) with direct connections to the vagal nucleus (Critchley et al., 2004). Sustained tongue-palate contact may be associated with shifts in trigeminal–vagal tone, with downstream effects on prefrontal activation and autonomic regulation.

### 4.3 Nostril dominance laterality: *svara yoga* and the nasal cycle

The classical *svara yoga* tradition (Śiva Svarodaya, 1984) systematically documents the alternation of nasal dominance and its association with different functional states. Modern respiratory physiology has confirmed the existence of the ultradian nasal cycle (Stoksted, 1953; Hasegawa and Kern, 1977)—a 2–4-h alternation of congestion and decongestion between the two nostrils—regulated by the hypothalamus.

The physiological correlates are directly relevant to this framework: Right nostril dominance (*piṅgalā*) is associated with increased sympathetic activation and left-hemisphere dominance; left nostril dominance (*iḍā*) is associated with parasympathetic dominance and right-hemisphere activation; and balanced bilateral breathing (*suṣumṇā svara*) is described in the classical texts as the condition required for kundalini awakening—a state the modern literature associates with autonomic equilibrium (Telles et al., 1994; Raghuraj et al., 1998).

We propose that the ratio of left:right nasal airflow (measured by rhinomanometry or peak nasal inspiratory flow) may serve as an additional biomarker at the Viśuddha (Pharyngeal plexus) station, with balanced bilateral flow (*suṣumṇā svara*) representing optimal autonomic equilibrium. This biomarker has the advantage of being non-invasive, continuously measurable, and directly grounded in both classical textual authority (*Śiva Svarodaya*; *Hatha Yoga Pradīpikā* II.10–11) and modern physiology. It is noted as a candidate biomarker requiring validation.

## 5 The fragmentation–integration continuum: a proposed biomarker framework

### 5.1 NDI construction and current developmental status

The NDI is an exploratory heuristic instrument at the earliest stage of development. It has not undergone external validation, reliability testing, psychometric evaluation, or normative data collection. It is presented in this article solely as a proof-of-concept framework to motivate and structure future validation research.

The NDI integrates seven measurable domains of nervous system function into a single 100-point composite index. Each domain is scored on a 0–100 sub-scale. For domains with established quantitative measures (HRV in the Autonomic domain, cortisol slope in the Neuroendocrine domain), the scoring uses published clinical thresholds. For domains relying on clinician assessment, the subjective component is explicitly acknowledged (see Appendix A). The composite NDI is computed as the weighted sum: NDI = *Σ*(wᵢ × Domainᵢ). These weights were derived from clinical observation, not from factor analysis or psychometric validation, and therefore independent validation is required.

A note on cardiac coherence and HRV: the Autonomic domain currently uses HRV (RMSSD) as its primary proxy. Cardiac coherence—defined as the ratio of peak power in the low-frequency band (0.04–0.15 Hz) to the sum of power in the low- and very-low-frequency bands (McCraty et al., 2009)—captures a related but distinct phenomenon: the regularity and sinusoidal quality of the heart rhythm pattern, reflecting the degree of synchronisation between sympathetic and parasympathetic branches. We recommend that future validation studies assess both HRV and cardiac coherence independently, as they may provide complementary information—HRV capturing overall autonomic tone and cardiac coherence capturing synchronisation quality.

The NDI may be positioned relative to existing instruments as follows. The KAS (Sanches and Daniels, 2008) captures subjective experiential dimensions without an underlying physiological model. The Physio-Kundalini Syndrome Index (Greyson, 1993) focuses on symptomatic presentations. Standard clinical instruments [Patient Health Questionnaire-9 (PHQ-9), Generalized Anxiety Disorder-7 (GAD-7), Pittsburgh Sleep Quality Index (PSQI), and Heart Rate Variability (HRV) indices] measure isolated parameters. The NDI’s proposed contribution is a multi-domain composite integration. This integration is the NDI’s theoretical contribution, not yet its empirical achievement.

### 5.2 The fragmentation cascade

The fragmentation cascade that is associated with State I-b (NDI > 60) is a well-documented physiological sequence. Chronic psychosocial stress activates the HPA axis, elevating cortisol. Sustained cortisol elevation is neurotoxic to hippocampal neurons (McEwen, 1998). Hippocampal volume reduction impairs prefrontal regulatory capacity. Amygdala hyperactivation increases threat-detection sensitivity, completing a self-reinforcing cascade (Thayer and Lane, 2009).

## 6 Preliminary clinical observations: the retrospective cohort dataset

The following section presents a retrospective observational dataset from a single-practitioner clinical setting. This is not a randomised controlled trial. No control arm, blinding, or randomisation was employed. Selection bias, single-practitioner confounding, and the absence of independent outcome verification are significant limitations. The data are presented as preliminary evidence sufficient to motivate formal prospective investigation, not as proof of efficacy. All results are hypothesis-generating.

### 6.1 Attachment-based sleep therapy (ABST): cohort description

Between 2010 and 2024, 404 consecutive patients presenting with chronic insomnia (sleep onset latency >30 min or total sleep time <6 h for >3 months with functional impairment, excluding primary sleep disorders on polysomnography) were offered attachment-based sleep therapy as a first-line intervention before pharmacological management. The ABST protocol draws on Winnicott’s concept of the transitional object (Winnicott, 1953) and Bowlby’s attachment theory (Bowlby, 1988). The protocol involves (a) identification of one emotionally meaningful object; (b) placement of this object in contact with the body during the pre-sleep transition; (c) maintenance of an intention of emotional receptivity rather than sleep-seeking; and (d) no sleep hygiene modifications in the initial 2 weeks.

Of 404 patients, 332 (82.2, 95% CI: 78.2–85.7%) met pre-defined improvement criteria, defined as: sleep-onset latency reduction to <15 min OR an increase in total sleep time to >6.5 h, sustained for >4 weeks without pharmacological intervention. Mean baseline sleep-onset latency was 58.3 ± 24.1 min and mean post-intervention: 11.7 ± 6.3 min. Mean baseline total sleep time was 5.1 ± 0.9 h and mean post-intervention in responders: 7.0 ± 0.7 h. Of 72 non-responders, 48 (11.9%) showed partial improvement with combined intervention, and 24 (5.9%) did not respond, of whom 19 subsequently received primary sleep disorder diagnoses.

Bias considerations. Six specific sources of potential bias: (1) selection bias; (2) single-practitioner confounding; (3) placebo and expectancy effects; (4) reporting bias; (5) absence of control condition; (6) absence of long-term follow-up data beyond the 4-week window. These figures are presented as observational data warranting formal RCT investigation.

### 6.2 The recursive self-inquiry protocol (RSIP)

The RSIP is a structured metacognitive intervention consisting of five stages (Interrupt → Observe → Interrogate → Exhaust → Recognise), with a typical session duration of 15–25 min per cycle, initial frequency of clinician-guided 2×/week transitioning to self-directed daily use (Vago and Silbersweig, 2012; Siegel, 2007).

## 7 The threshold state: neuroimaging evidence, phenomenology, and research horizons

State III—threshold coherence—is the most epistemically demanding component of the theoretical framework, drawing on neuroimaging research, cross-tradition phenomenological consistency, and first-person observational data.

### 7.1 Neuroimaging evidence for non-ordinary contemplative states

Lutz et al. (2004) documented high-amplitude gamma synchrony (40 Hz+) in Tibetan Buddhist practitioners. Brewer et al. (2011) demonstrated meditation-associated alterations in default mode network activity. Josipovic (2014) research on non-dual awareness identified neural correlates distinct from focused attention meditation. Berkovich-Ohana et al. (2012) documented mindfulness-induced changes in gamma activity.

### 7.2 Cross-tradition phenomenological consistency

The structural consistency of the threshold state’s description across traditions operating within substantially different frameworks is striking. The Yoga Sūtras describe *kaivalya*, Buddhist *nirodha-samāpatti* describes cessation, Advaita’s *mokṣa* describes non-dual recognition, and Stoic *apatheia* describes freedom from disturbance. While significant intra-South-Asian cross-pollination is historically well documented, the convergence between South Asian and Greek traditions offers a stronger case for independent observation of a common phenomenological structure (Patanjali, 2012 c. 400 CE).

## 8 The R-operator: a mathematical framework for liberation phenomenology

The R-Operator formalism is developed in full in the companion article (Samanta, 2026, *Liberation Mathematics I*, accepted, *Frontiers in Psychology*). The Liberation Operator: L(t) = TRUE if and only if R(M, M’) is continuously computed for all encounters. Full derivation, cross-tradition convergence proofs, and falsifiable predictions are presented in the companion article.

## 9 Method protocols: from fragmentation to integration

The 8-week sequential integration protocol addresses the proposed fragmentation cascade in reverse order, moving bottom-up through the seven candidate plexus stations, consistent with the polyvagal hierarchy (Porges, 2011) and classical bottom-up description of kundalini movement in the texts (Svātmārāma, 1974). Safety parameters include the following: progression rate >5% NDI improvement per week treated as a signal to reduce practice intensity; contraindications for active psychosis, bipolar I in active phase, cardiovascular instability, and acute trauma without concurrent therapeutic support.

## 10 Discussion

### 10.1 The Iconogram as a research programme

This article has proposed that the Shiva-serpent iconographic complex may encode a three-state model of nervous system organisation whose correspondence with contemporary neuroscience is sufficiently precise to constitute a research programme. The comparable precedent: Harvey’s description of circulation drew on systematic observation without any understanding of the mechanism; Jenner’s vaccination encoded empirical observations before germ theory existed.

### 10.2 Convergence with contemporary consciousness research

The threshold state finds its most direct parallels in non-dual awareness research (Josipovic, 2014), ego dissolution research (Newberg and Iversen, 2003), and DMN self-referential processing (Brewer et al., 2011). The R-Operator formalism may provide a mathematical complement to the S-ART framework (Vago and Silbersweig, 2012).

### 10.3 Epistemological constraints: the limits of analogical reasoning in cross-paradigm research

First, confirmation bias. When a researcher is simultaneously the clinician, the theorist, and the practitioner, the risk of unconscious filtering through the theoretical framework is substantial. This is the single strongest argument for independent replication.

Second, the limits of structural analogy. Structural isomorphism can be coincidental. The proposed correspondences are candidate hypotheses requiring direct empirical testing.

Third, interpretive overdetermination. Rich symbolic systems are sufficiently complex that multiple frameworks can be mapped onto them. Our neurophysiological reading is one of several possible systematic readings. Its value lies in generating falsifiable predictions testable independently of the interpretive framework.

## 11 Limitations and future directions

Several limitations require acknowledgement. First, the observational cohort is not an RCT. Second, the NDI is an exploratory heuristic metric (see Section 4.1). Third, the chakra-to-plexus mapping requires systematic empirical testing. Fourth, State III remains a theoretical proposition. Fifth, the iconographic interpretations are proposed as phenomenological correspondences, not claims about historical intent.

Future Research Directions. Priority 1: Independent NDI validation against KAS, PHQ-9, GAD-7, PSQI, and HRV indices. Priority 2: Multi-centre RCT of the eight-week protocol. Priority 3: Neuroimaging study pre-protocol and post-protocol. Priority 4: Cross-tradition survey using R-Operator coding. Priority 5: Concurrent NDI and KAS subscale validity testing. Priority 6: Nostril dominance laterality validation as an autonomic equilibrium biomarker (Telles et al., 1994). Priority 7: Cardiac coherence as a complement to HRV within the NDI Autonomic domain (McCraty et al., 2009).

## 12 Conclusion

This article has proposed that the Shiva-serpent iconographic complex may encode a three-state model of nervous system organisation whosze correspondence with contemporary clinical neuroscience is sufficiently coherent to constitute a testable research programme. The model generates four specific falsifiable predictions. The preliminary retrospective clinical observations—Ethics approval HP/EC/APVL/20–24/058.3—provide exploratory support for the integration framework. The Shiva-serpent iconographic complex, read as a candidate phenomenological map, demonstrates a degree of structural correspondence with contemporary neurophysiological models that warrants systematic empirical investigation.

## Data Availability

The raw data supporting the conclusions of this article will be made available by the authors, without undue reservation.

## Supplementary material

SUPPLEMENTARY FIGURE S1 Shiva-serpent iconographic reference showing three positional states of Vāsuki encoding the proposed neurophysiological correspondences. (a) State I — Kundalinī Śayitā: serpent coiled at base. (b) State II — Kundalinī Jāgrtā: serpent ascending. (c) State III — Kundalinī Muktā: serpent at neck in 360° loose topology with visible gap. [Schematic reconstruction; not derived from specific archaeological sources].

## Appendix AObservational procedures and documentation standards

### Setting

All observations were recorded in a single-practitioner outpatient clinical setting specialising in autonomic dysregulation, sleep disturbance, and attentional dysfunction.

### Documentation

All [CLINICALLY OBSERVED] claims were documented in contemporaneous clinical notes at the time of each patient encounter, not reconstructed retrospectively.

### Classification criteria

[MEASURED]: Supported by published literature or quantitative cohort data. [CLINICALLY OBSERVED]: Documented systematically across multiple patients but not RCT-validated. [THEORETICAL MODEL]: Proposed frameworks without direct empirical support.

### Limitations

Single-practitioner setting precludes independent verification. No inter-rater reliability. Self-report outcomes without blinded assessment. Consumer-grade HRV devices. Actigraphy-estimated sleep efficiency. These motivate the prospective validation studies proposed in Section 10.
