# Supplementary figures and images for: Towards a neurophysiological model of kundalini: a theoretical framework informed by preliminary clinical observations
Source: Front Behav Neurosci. 2026 Jun 10;20:1828520. doi: 10.3389/fnbeh.2026.1828520 (PMC13291124; doi:10.3389/fnbeh.2026.1828520)

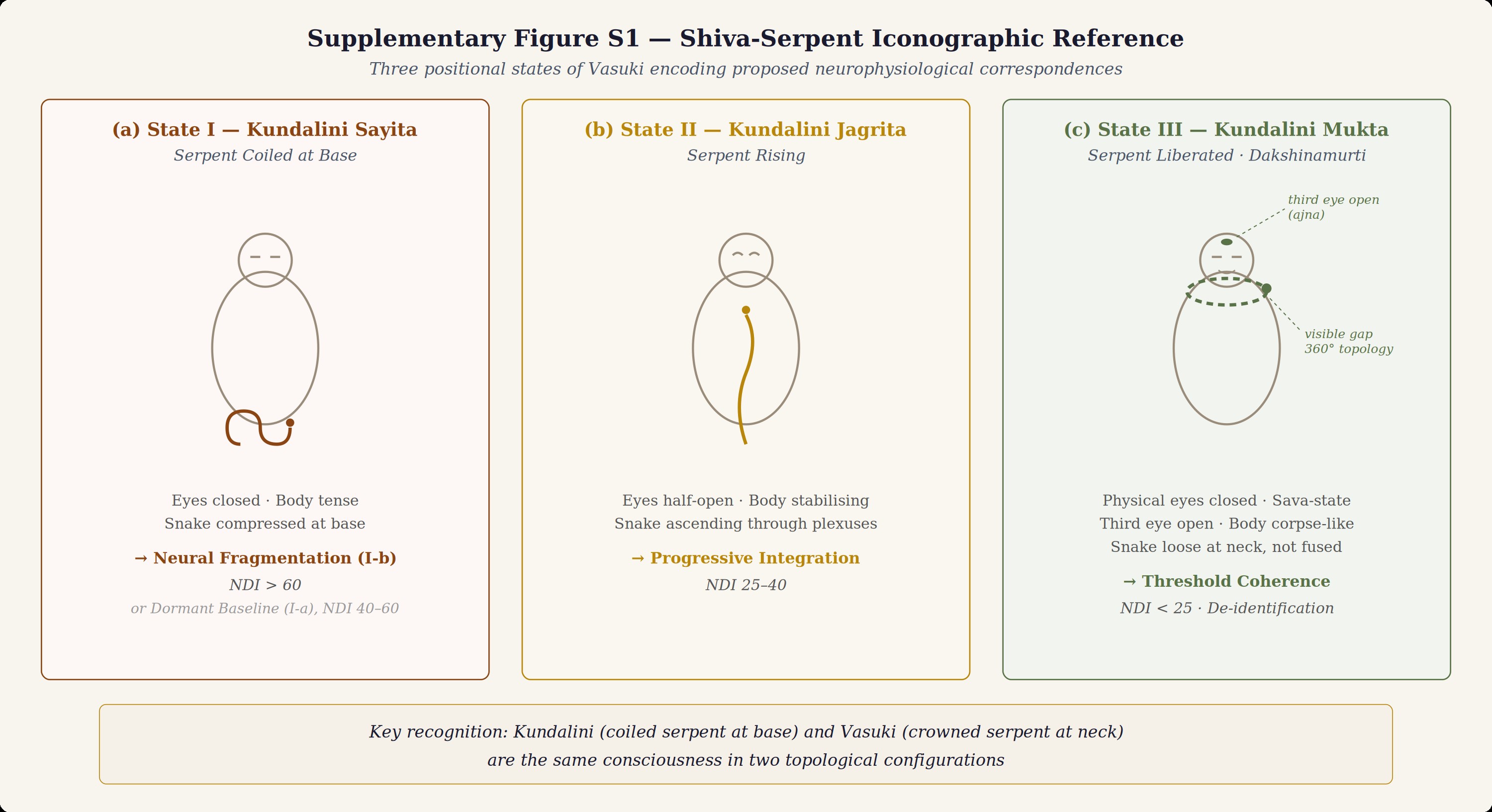

Supplement: SUPPLEMENTARY FIGURE S1 — Shiva-serpent iconographic reference showing three positional states of Vāsuki encoding the proposed neurophysiological correspondences. (a) State I — Kundalinī Śayitā: serpent coiled at base. (b) State II — Kundalinī Jāgrtā: serpent ascending. (c) State III — Kundalinī Muktā: serpent at neck in 360° loose topology with visible gap. [Schematic reconstruction; not derived from specific archaeological sources]. [file Image_1.jpg]
