# Supplementary material for: Towards a neurophysiological model of kundalini: a theoretical framework informed by preliminary clinical observations
Source: Front Behav Neurosci. 2026 Jun 10;20:1828520. doi: 10.3389/fnbeh.2026.1828520 (PMC13291124; doi:10.3389/fnbeh.2026.1828520)
Supplement: Supplementary file 2 [file Supplementary_file_1.docx]

**SUPPLEMENTARY MATERIAL**

*Toward a Neurophysiological Model of Kundalini:*

*A Theoretical Framework Informed by Preliminary Clinical Observations*

Samanta S*, Sultania N, Roychoudhury M, Sharma S, Mitra P

Manuscript ID: 1828520 · Frontiers in Behavioral Neuroscience

# **Supplementary Table S1**

**ABST Cohort Demographics and Outcomes Summary (n = 404)**

*Retrospective observational data, single-practitioner setting, 2010–2024. No randomisation or control arm. All values are descriptive. Ethics approval: HP/EC/APVL/20–24/058.3.*

| **Parameter** | **Value** | **Notes** |
| --- | --- | --- |
| ***Demographic Characteristics*** |  |  |
| **Total cohort** | n = 404 | Consecutive patients |
| **Age range** | 18–72 years |  |
| **Mean age (± SD)** | 38.7 ± 12.4 years |  |
| **Gender (Female : Male)** | 242 : 162 (59.9% : 40.1%) |  |
| **Median duration of insomnia** | 4.2 years (IQR: 2.1–8.6) | Self-reported |
| **Comorbid anxiety (clinical dx)** | 168 (41.6%) | Formal diagnosis on record |
| **Comorbid depression (clinical dx)** | 112 (27.7%) | Formal diagnosis on record |
| **Prior pharmacological tx** | 276 (68.3%) | Any prior sleep medication |
| **Prior non-pharma tx attempted** | 184 (45.5%) | CBT-I, sleep hygiene, etc. |
| ***Baseline Metrics*** |  |  |
| **Sleep-onset latency (mean ± SD)** | 58.3 ± 24.1 min | Self-reported sleep diary |
| **Total sleep time (mean ± SD)** | 5.1 ± 0.9 hours | Wrist actigraphy estimate |
| **HRV RMSSD (mean ± SD, n=287)** | 31.4 ± 11.2 ms | Consumer-grade wearable |
| **Baseline NDI (mean ± SD)** | 64.2 ± 9.8 | Clinician-assessed heuristic |
| ***Outcomes at 4 Weeks*** |  |  |
| **Responders (met criteria)** | 332 (82.2%) | 95% CI: 78.2–85.7% |
| **Post-intervention SOL (responders)** | 11.7 ± 6.3 min | Δ52.6 min reduction |
| **Post-intervention TST (responders)** | 7.0 ± 0.7 hours | Δ1.9 hours increase |
| **Partial responders** | 48 (11.9%) | Improved with combined tx |
| **Non-responders** | 24 (5.9%) |  |
| **Primary sleep disorder (dx on PSG)** | 19 (4.7%) | Among non-responders |
| **Post-intervention NDI (responders)** | 47.3 ± 8.1 | Δ16.9 points |
| **Post-intervention HRV (n=287)** | 42.8 ± 9.7 ms | Δ11.4 ms increase |
| ***Response Rate by Baseline Severity*** |  |  |
| **Mild insomnia (SOL 30–45 min)** | 94.2% (n=121) |  |
| **Moderate insomnia (SOL 45–75 min)** | 83.7% (n=196) |  |
| **Severe insomnia (SOL > 75 min)** | 62.1% (n=87) |  |

*All outcomes are self-reported or clinician-assessed; not independently verified. HRV available for 287/404 patients (71.0%) who had compatible wearable devices. NDI is an exploratory heuristic metric (see main text Section 4.1). Response defined as SOL < 15 min OR TST > 6.5 hours, sustained > 4 weeks without pharmacological intervention.*

# **Supplementary Table S2**

**Eight-Week Sequential Integration Protocol: Week-by-Week Detail**

*Bottom-up plexus-targeted intervention consistent with polyvagal hierarchy (Porges, 2011). Safety thresholds: progression rate > 5% NDI improvement/week signals reduced intensity. [THEORETICAL MODEL + CLINICALLY OBSERVED]*

| **Weeks** | **Target Station** | **Breathing / Attentional** | **Somatic / Postural** | **Candidate Biomarkers** | **Expected NDIΔ** |
| --- | --- | --- | --- | --- | --- |
| **1–2** | **Mūlādhāra Sacral (S2–S4)** | Body scan (20 min/day) Earth-walking (20 min/day) Pelvic floor awareness Grounding visualisation | Mula Bandha (gentle) Supine root poses Contact with floor/earth | Pelvic floor EMG Gut serotonin Self-report: safety | NDI Δ53–5% HRV: +2–4 ms “Grounded” |
| **2–3** | **Svādhiṣṭhāna Hypogastric** | Abdominal breathing (6/min) Uddiyana Bandha intro ABST transitional object | Hip-opening postures Abdominal massage Emotional journalling | DHEA/cortisol ratio Self-report: emotion | NDI Δ53–4% Sleep improving “Flowing” |
| **3–4** | **Maṇipūra Celiac (T5–T12)** | Kapalabhati (5 min) Cortisol-targeting breathwork RSIP for 3 reactive episodes | Nauli kriya (gentle) Abdominal core work Heat-building practices | Cortisol diurnal rhythm Insulin sensitivity Gut motility | NDI Δ54–6% Cortisol slope “Empowered” |
| **4–5** | **Anāhata Cardiac (T1–T5, X)** | Nadi Shodhana (10 min) Kumbhaka (gentle, with caution) Compassion cultivation | Heart-opening postures Celiac plexus activation Lingual exercises (5 min) | HRV (RMSSD) Cardiac coherence Oxytocin (proxy) | NDI Δ55–7% HRV > 40 ms “Open heart” |
| **5–6** | **Viśuddha Pharyngeal (IX, X, XI)** | Ujjayi pranayama (15 min) Khechari Mudra practice Vocalisation exercises | Jalandhara Bandha Neck/throat stretches Nostril dominance tracking | Thyroid profile Nostril laterality Upper vagal tone | NDI Δ53–5% Balance emerging “Voice freed” |
| **6–7** | **Ājñā PFC–Pineal axis** | Trataka (candle gazing, 10 min) Third-eye dhāraṇā R-Operator journalling | Shambhavi Mudra Forward bends w/ awareness Dark-room practice | Alpha coherence Melatonin (proxy) PFC engagement | NDI Δ53–4% Focus > 10 min “Seeing clearly” |
| **7–8** | **Sahasrāra Whole-brain (DMN)** | Open awareness (20 min) R-Operator in vivo encounters Reduce formal practice | Meditation without object Integration walks Maintenance protocol design | Gamma coherence DMN deactivation Global NDI | NDI Δ52–3% NDI target < 40 “Oneness” |

*All expected NDI changes are observational estimates from the clinical cohort; not validated benchmarks. Contraindications: active psychosis, bipolar I (active phase), cardiovascular instability, acute trauma without concurrent support. Kumbhaka requires caution with hypertension, glaucoma, or arrhythmia.*

# **Supplementary Table S3**

**Neural Dispersion Index (NDI): Detailed Domain Scoring Rubric**

*Exploratory heuristic instrument. No external validation, reliability testing, or psychometric evaluation has been conducted. Presented as proof-of-concept only. See main text Section 4.1.*

| **Domain** | **Weight** | **Measurement Proxy** | **Fragmented (Score 60–100)** | **Transitional (Score 30–60)** | **Integrated (Score 0–30)** |
| --- | --- | --- | --- | --- | --- |
| **Autonomic** | **15%** | HRV (RMSSD) Consumer wearable | RMSSD < 25 ms: Score 80–100 RMSSD 25–35: Score 60–80 Flat diurnal pattern | RMSSD 35–50 ms: Score 40–60 Mild diurnal variation Occasional dysregulation | RMSSD > 50 ms: Score 0–40 Clear diurnal pattern Stable parasympathetic tone |
| **Neuroendocrine** | **15%** | Cortisol diurnal slope Clinician assessment | Flat cortisol curve (AM ≈ PM) Elevated evening cortisol HPA axis non-responsive | Mild morning peak emerging Partial rhythm restoration Stress response still elevated | Clear AM peak, PM nadir Healthy cortisol awakening Appropriate stress response |
| **Attentional** | **15%** | Sustained focus duration Self-reported + clinical | Focus < 3 min without break Constant mind-wandering Unable to complete tasks | Focus 3–8 min, variable Some ability to redirect Task completion improving | Focus > 12 min sustained Voluntary attention control Task completion consistent |
| **Somatic** | **10%** | Body coherence Clinician assessment | Chronic pain, dissociation Poor proprioception Somatisation symptoms | Moderate body awareness Pain present but managed Improving interoception | Stable body awareness Pain observed, not fused Full proprioceptive clarity |
| **Affective** | **15%** | Emotional regulation Clinician + self-report | Highly reactive, labile Emotional flooding common Poor frustration tolerance | Moderate stability emerging Less reactive, some flooding Improving resilience | Responsive, not reactive Emotional range preserved Rapid recovery from stress |
| **Cognitive** | **15%** | PFC engagement Clinical + task-based | Executive function impaired Poor planning/organisation Decision paralysis | Moderate exec function Some planning ability Decisions with support | Sustained executive function Flexible planning Independent decisions |
| **Interpersonal** | **15%** | Social coherence Clinician + self-report | Social withdrawal/disconnect Conflict-driven relating Attachment dysregulation | Variable social engagement Some secure interactions Conflict reducing | Secure, attuned relating Empathic engagement Healthy boundaries |

*NDI composite = Σ(wᵢ × Domainᵢ). Weights derived from clinical observation, not factor analysis. State classification: I-b (NDI > 60), I-a (40–60), II (25–40), III (< 25). For domains relying on clinician assessment, inter-rater reliability has not been established.*

# **Supplementary Table S4**

**Illustrative Single-Case Integration Trajectory (n = 1)**

*Patient: Female, 34 years, presenting NDI 67 (State I-b). HRV measured by RMSSD using consumer-grade wearable. Sleep efficiency actigraphy-estimated. All values self-reported except HRV. This single case is presented for clinical illustration only. No causal inference is warranted. [CLINICALLY OBSERVED — illustrative case only]*

| **Timepoint** | **NDI** | **HRV (RMSSD)** | **Cortisol Rhythm** | **Sustained Focus** | **Sleep Eff. *** | **Primary Interventions** |
| --- | --- | --- | --- | --- | --- | --- |
| **Baseline** | 67 | 28 ms | Flat | 3.1 min | 61% | — |
| **Weeks 1–2** | 58 | 33 ms | Mild slope | 4.8 min | 53% | Root grounding; body scan; earth-walking 20 min/day |
| **Weeks 3–4** | 51 | 39 ms | Emerging | 6.2 min | 47% | Abdominal breathing; Uddiyana Bandha; ABST object; RSIP |
| **Weeks 5–6** | 44 | 47 ms | Clear rhythm | 8.9 min | 39% | Nadi Shodhana; Kumbhaka; lingual exercises; celiac activation |
| **Weeks 7–8** | 41 | 54 ms | Strong rhythm | 11.4 min | 31% | Heart-brain integration; compassion; Mula Bandha; R-Operator |
| **3-Month F/U** | 38 | 58 ms | Robust | 13.1 min | 27% | Maintenance 2×/week; monthly NDI self-assess; RSIP daily in vivo |

** Sleep Efficiency is defined as (Total Sleep Time / Time in Bed) × 100. The inverse relationship with NDI improvement reflects reduced sleep-onset latency and reduced wakefulness after sleep onset, while total sleep time was maintained. Lower sleep efficiency percentage here paradoxically reflects healthier sleep: less time awake in bed.*

*Integration rate: Week 0→2 = 8.8%/week (⚠️ above 5% safety threshold — intensity was reduced); Week 2→4 = 3.8%/week (✓ optimal); Week 4→8 = 2.4%/week (✓ safe).*

# **Supplementary Table S5**

**Cross-Tradition Liberation Phenomenology: R-Operator Structural Comparison**

*The R-Operator (Relativity Operator) is defined as: R(M, M’) = [M meets M’] → [neither M nor M’ remains position-fixed]. This table maps the functional principle across seven contemplative traditions. Full derivation in Samanta (2026), Liberation Mathematics I, accepted, Frontiers in Psychology. [THEORETICAL MODEL]*

| **Tradition** | **Liberation Term** | **R-Operator Expression** | **Body State Description** | **Iconographic Completeness** | **Primary Source** |
| --- | --- | --- | --- | --- | --- |
| **Yoga (Pātañjala)** | **Kaivalya** | Isolation of pure awareness from identifications with prakrti | Body in stillness; two eyes closed; third eye open; serpent crowned | ✓ Full: both body state and consciousness state depicted iconographically | Yoga Sūtras IV.34 (Patanjali, c. 400 CE) |
| **Buddhism (Theravāda)** | **Nirodha-samāpatti** | Cessation of perception and feeling; consciousness present but unattached | Meditation posture; eyes closed or downcast; minimal body description | ⚠️ Partial: consciousness state described; body state less specified | Viṣuddhimagga XXIII (Buddhaghosa, c. 5th CE) |
| **Advaita Vedānta** | **Mokṣa / Nirvikalpa Samādhi** | Recognition of non-dual nature of consciousness; Ātman = Brahman | Formless absorption; text-based, not iconographic | ⚠️ Partial: consciousness state described; body state in texts only | Vivekacūḍāmaṇi (Śaṅkarācārya) |
| **Jainism** | **Kayotsarga / Kevalajñāna** | Omniscient, detached; standing or sitting motionless | Standing motionless; fasting; complete detachment | ⚠️ Partial: posture and detachment shown; not full consciousness map | Tattvārtha Sūtra (Umāsvāti, c. 2nd CE) |
| **Tibetan Buddhism** | **Thukdam (clear light)** | Clear light consciousness at death; clinical death, body preserved | Body in death-like state; warm; not decomposing | ✓ Full: both body state (death-like) and consciousness state (clear light) | Bardo Thödol (c. 8th CE) |
| **Greek Stoicism** | **Apatheia / Ataraxia** | Freedom from disturbance; ruling faculty no longer buffeted by passions | No specific body iconography; philosophical description only | ❌ Minimal: functional principle described; no body state or icon | Discourses (Epictetus, c. 108 CE) |
| **Daoism** | **Zuowang (sitting-forgetting)** | Forgetting the body, forgetting knowledge; merging with Tao | Sitting; minimal physical specification | ⚠️ Partial: sitting posture; forgetting process described | Zhuangzi Ch. 6 (c. 3rd century BCE) |

*The Shiva-serpent iconographic complex is unique in providing both body-state and consciousness-state information simultaneously in a single visual representation. Intra-South-Asian cross-pollination (Buddhist ↔ Pātañjala ↔ Vedantic) is historically well documented; the convergence between South Asian and Greek/Daoist traditions offers stronger evidence for independent observation. See main text Section 6.2.*

# **Supplementary Table S6**

**Recursive Self-Inquiry Protocol (RSIP): Five-Stage Detail**

*Structured metacognitive intervention for attentional dysregulation, compulsive behaviour, chronic reactivity, and generalised anxiety. Proposed mechanism: prefrontal activation over limbic circuits through sustained recursive engagement. [CLINICALLY OBSERVED; n = 404; see Appendix A]*

| **Stage** | **Objective** | **Clinician Instruction** | **Phenomenological Marker** | **Clinical Notes** |
| --- | --- | --- | --- | --- |
| **1. INTERRUPT** | Recognise that a reactive pattern has been activated. Stop the automatic response chain before it completes. | Clinician prompt: “Notice that you are reacting.” Patient learns to catch the impulse before action. | Reactive chain halted mid-sequence. Physical pause (2–5 seconds). Awareness of body state. | 2–3 sessions to learn; patient may initially miss entirely |
| **2. OBSERVE** | Direct attention to the reactive state itself — not its content, but its presence as a phenomenon in awareness. | “What is happening in your body right now? Where is the sensation? What is its quality?” | Shift from content to process. Body-level awareness of emotion. Naming without narrating. | Often produces initial anxiety increase (normal) |
| **3. INTERROGATE** | Recursively question the identity structures that generated the reaction. “Who is reacting? What does this reaction protect?” | Sustained questioning (5–10 min) without accepting the first answer. Each answer is questioned further. | Identity assumptions begin to surface. Patient recognises “I am angry” vs. “Anger is present.” | Most intellectually challenging stage; patients may resist |
| **4. EXHAUST** | Continue recursive questioning until the reactive pattern runs out of fuel. No new content is generated. | “Keep looking for who is angry. Keep looking for who needs to be right.” | Silence. Stillness. The reactive pattern dissolves without resolution — it simply stops. | Key clinical milestone: the point of cessation is observable |
| **5. RECOGNISE** | In the silence after exhaustion, notice what remains. Awareness itself is present, without content. | “What is here now, when the reaction has stopped?” Patient directed to awareness, not content. | Witness state. Awareness without object. Often described as “spacious” or “empty but present.” | May last seconds initially; extends with practice |

*Typical session: 15–25 min per cycle. Initial frequency: clinician-guided 2×/week. Progression to self-directed daily use when patient can reach Stage 4 without external prompting. Progression criteria: demonstrated ability to reach “Exhaust” independently.*
